# Treading the clinical pathway: a qualitative study of advanced practice nurses in a local health district in Australia

**DOI:** 10.1186/s12912-015-0105-7

**Published:** 2015-10-19

**Authors:** Lauretta Luck, Lesley Wilkes, Jennifer O’Baugh

**Affiliations:** Curriculum Renewal- Senior Lecturer, School of Nursing & Midwifery, University of Western Sydney, Locked Bag 1797, Penrith, South DC NSW 2751 Australia; School of Nursing and Midwifery, University of Western Sydney/Nepean Blue Mountains Local Health District, Kingswood, Australia; Centre for Nursing Research and Practice Development, Nepean Hospital, PO Box 63, Penrith, NSW 2751 Australia; Nepean Blue Mountains Local Health District, Nepean Hospital, Penrith, NSW 2751 Australia; Centre for Nursing Research and Practice Development, Nepean Blue Mountains Local Health District, Nepean Hospital, P.O. Box 63, Penrith, 2751 NSW Australia

**Keywords:** Nurse, Nursing, Career planning, Qualitative research, Clinical nurses

## Abstract

**Background:**

Career planning in nursing is often haphazard, with many studies showing that nurses need personal motivation, education, and the support of workplaces, which are often dominated by political and fiscal agendas. Nurses often need institutional and personal support to plan their careers and make decisions regarding their career aspirations.

**Method:**

A descriptive qualitative design was used. Data were gathered using semi-structured digitally recorded interviews and analysed for common categories. Twenty seven (*n* = 27) participants were interviewed.

**Results:**

There were four categories revealed by the participants who described their career progression experiences: moving up the ladder, changing jobs for career progression, self-driven and the effects of institutional environments.

**Conclusion:**

Many of the participants’ careers had been shaped serendipitously. Similar to other studies, these nurses felt political, institutional and financial factors impacted on their career opportunities. There are implications for nursing managers with more support required for nurses to plan their career trajectories. In addition to an organisation centred approach to career planning, nurse leaders and managers must take into account the personal and professional requirements of their nurses. Nurses themselves also need to take personal responsibility for career development. Greater support for nurses’ career planning and personal drive will help organisations to plan their future workforce needs.

## Introduction

Increasingly, nurses working in clinical roles are becoming more educated at postgraduate levels. In 2008, there were over 160 masters programs and 16 doctoral programs for clinical nurses listed in Nursing Programs in the United States of America (USA) [1]. Similarly in Australia, all 37 universities offering nursing conduct clinically focused Masters and Doctoral degrees [2]. As nurses position themselves in the second decade of the 21^st^ century where a multiple career is considered the norm and nurses have a higher degree level of education, it is important that nurses pay heed to their career development and planning. As Styles [3] stated, in the early 1980s, “the professionalism of nursing will be achieved only through the profession-hood of its members” (pp. 8). One way of guiding nurses in career development is through an understanding of how people tread a particular path to success and professionalism in nursing. This paper focuses on the personal experiences of advanced practice nurses in an Australian health service area and their varied paths to their current positions.

## Background

New roles for clinical nurses “who provide expert clinical practice, facilitating change, disseminating evidence based practice and improving communication in and beyond the health team” [4] have developed over the past two decades with a myriad of titles including clinical nurse specialists, clinical nurse consultants, clinical nurse leaders and nurse practitioners. Confusion exists across countries in the Western World as to what differences these roles and titles signify [5]. Specialist nurses who are commonly given the title of Clinical Nurse Specialist (CNS) in the USA, United Kingdom and some States in Australia [6]. Wilkes et al. [7] focus on specific defined areas of nursing [5]. However, in the context of this study in New South Wales (NSW), Australia, there are three levels of expertise or advanced practice: the CNS, Clinical Nurse Consultant (CNC) and Nurse Practitioner (NP). While in NSW, Australia, CNS, CNC and NP are called advanced practice nurses, this is not always the case in other countries where there is delineation between specialist nurses and advanced practice nurses. Commonly, in the USA, these NPs provide primary care [5].

In NSW, Australia, the CNS is a registered nurse who has a postgraduate qualification and also has twelve months experience post registration or four years’ experience in the nursing specialty. The main emphasis in the role is clinical bedside practice. The CNC is a nurse with five years’ experience, and postgraduate qualifications in the nursing speciality preferably at Masters level. The main role functions of the CNC are: clinical leadership, clinical service and consultancy, clinical planning and management, research and education [7]. The functions are closely aligned to the United Kingdom “Link Nurse” role, where the nurse acts as a link between theory and practice [8]. In the context of this study, a NP is a nurse approved by the Director-General of NSW Health [9], who must have a Masters level qualification for a Nurse Practitioner and has completed a broad range of healthcare services that may include autonomous and independent clinical decision making [10].

In exploring the pathways that clinical nurses take during their career, it is important to explore the concept of career planning and models used in nursing to encourage career development. Career planning is “a continuous process of self-assessment and goal setting” [11], which can occur in a closed or open system [11]. In the closed system, the organisation assesses future directions, estimates human resource needs, defines job parameters, seeks and selects candidates, appraises employees, and provides on-the-job training. The open system includes the above characteristics but also incorporates staff appraisal of their work-life experiences and career aspirations, assessment of their own competencies and interests, exploration of opportunities and then development of their career plans.

There are a number of models and theories on factors influencing and assisting career planning and development that have been used by a myriad of nurse researchers. For example, Duffield et al. [12] utilised the Kanter [13] model of structural power and undertook a project focused on job enrichment and enhancement of nurses’ personal empowerment. They found this provided useful career development opportunities for experienced nurses by allowing them to undertake work of a more strategic nature rather than direct clinical care with the intention of broadening their experience and preparing them for more senior roles within the bounds of organisational needs. Other models for career planning included the PECAN framework of Adeniran et al. [14] which emphasised system factors, social and human capital factors, external support and professional preparedness. Further, Donner and Wheeler [15] emphasised the process of career progression and identified a five stage career planning and development model. The stages included the nurses understanding their environment, undertaking self-assessment, developing a career vision, developing a career plan and marketing themselves. This model emphasised the fact that career development is iterative rather than a linear process with the individual needing to take responsibility for career development. Using a system theory framework, Patton and McMahon [16] identified that career advancement can be influenced by individual characteristics, such as gender, past and present events in individual lives, and that the social and political environment of the workplace also impacts upon individual career trajectories. From these and other studies in nursing, the two key motivators for individual decision-making regarding career progression are professional empowerment [17, 18] and job satisfaction [12, 15, 19, 20].

Aligning personal strengths with passions helps individuals gain momentum in their career [21]. Shirey [22] advocates that nurses be paid to do things they love to do. There is a need for institutions, individual nurses and professional organisations to work together to support career development [15]. The most widely discussed mechanism to support nursing career development is through mentoring programs [14]. Shermont et al. [23] suggest that mentoring is essential to assist nurses with engagement in career mapping and to supporting the identification and development of future clinical practice leaders. The three year career planning program developed by Hall et al. [24] encouraged participants to scan their work environment, assess themselves in relation to their values, knowledge, strengths and limitations, and develop clear goals. Over the course of the program, they found that the nurses could develop a clear vision and consequently construct an individual career. The program improved work satisfaction, relationships with colleagues and work life balance [24].

An extensive review of the literature on career development and pathways for expert/specialist/advanced practice nurses was conducted using CINAHL, Google Scholar, Medline and ProQuest, but no studies were found that provided personal accounts and experiences of these nurses’ career trajectories. Identifying how and why these nurses moved into increasingly advanced roles, supports the careers of other nurses wishing to pursue similar roles in a dynamic ever changing health system and will assist with broader workforce planning processes both nationally and internationally. The aim of this study was to describe the career paths of a group of Clinical Nurse Specialists (CNS), Clinical Nurse Consultants (CNC) and Nurse Practitioners (NP) in a local health district in New South Wales.

## Method

A qualitative descriptive design was used in this research, which was chosen because it is considered the best method when little is known about the phenomenon of interest as in this study [25]. This research design acknowledges the participants’ subjective experiences and enables insights into how individuals understand and construct their lives [26]. Importantly, these descriptions are accounts of everyday lives. The findings describe self-reports of advanced practice nurses’ career pathways in an Australian context.

### Sample

The sample consisted of advanced practice nurses working as CNSs, CNCs and NPs who were employed within an urban local health district in New South Wales, Australia. The sample was self-selecting and accessed using publically available email lists. Flyers advertising the study were displayed in wards, units and community areas. We recruited until we had reached data saturation for each group (when the researchers could not see any new information from the interviews).

### Data collection

Qualitative in-depth semi-structured interviews were conducted with the participants by two members of the team and each took 20 – 45 min. All face-to-face or telephone interviews were digitally recorded. Interview questions related to the nurse participant’s career pathways, trajectories and choices since graduating. Demographic information including gender, age, specialty and number of years in their current role was also collected.

### Data analysis

The interviews were transcribed verbatim, using a transcription service. Two members of the research team coded and sorted the data. The transcribed text was coded for common and contrasting descriptions of nurses’ pathways to their current positions [26]. These descriptions were then sorted into four categories, which were confirmed by the members of the team. An audit trail was also kept during analysis to add to credibility and therefore ensure integrity and accuracy of the data and analysis processes. At the same time, quantitative data related to demographics were tallied, means and ranges were calculated and the results tabulated.

### Ethics

The project was approved by the relevant Local Health District Human Research Ethics Committee and the Human Research Ethics Committee of the University. Participants were given an information sheet explaining the research and what their participation would entail before they gave their written voluntary informed consent. No identifying details are reported in this paper, and only participant codes are used with exemplars.

## Results

### Participants

The demographic data of the participants are shown in Table 1. As indicated by the table, the majority of the participants were female, and there was little variation in overall experience or number of years in their current position.

**Table 1:** Demographic details of participants

Characteristics	CNS	CNC	NP
Age range	28–57	33–57	38–57
Gender	9 females, 1 male	9 females, 1 male	6 females, 1 male
Years in current position - range	0.5–10	1–10	0.6–8
Years of clinical experience- range	7–36	11–41	17–28

Reviewing the qualifications of participants, we can see all of the CNS participants had postgraduate certificates and five had relevant Master degrees. Of the CNC cohort, all but one participant had a postgraduate certificate and five of the participants had relevant Master degrees. All of the NPs had specialist Nurse Practitioner Masters, with one having four (4) Master degrees and another having two (2).

### Career pathways

The four categories that emerged from the analysed text to describe the nurses’ career progression experiences were: moving up the career ladder, changing jobs for career progression, self-driven, and the effects of institutional environments.

### Moving up the career ladder

The findings revealed that there were three pathways to move up the career ladder: First, a personal application for a position; second, an application for an advertised position; and third, fortuitously being given a secondment opportunity that led to promotion.

#### Personal application

In NSW Australia, career progression from a RN to a CNS is a personal decision based on fulfilling a set of criteria. This is illustrated in the following nurse’s experience:

*Just demonstration of the quality roles that I do, like, the auditing and local procedural development and mentoring of students, and postgraduate qualifications* (CNS 20).

#### Application for a position

Advancement to a CNC or NP position is via advertisement and appointment. While the CNCs were confident they met the criteria and had the prerequisite knowledge and experience, they were often hesitant to apply. Some were encouraged by nurses they worked with, others decided undertaking the application procedure would be a good experience for them.

*I wasn’t looking for a job, but I just thought, oh, I have never seen that CNC position vacant … and I know about every single aspect in that job description … so I rung up and I said, look, this is my experience, anyway she said, yeah, apply for it … so I applied for it (CNC 32).*

#### Secondment

Another way nurses moved up the career ladder was secondment, and these could be haphazard.

*I was either in the right place at the right time, or the wrong place at the wrong time. I happened to be walking past an office and they called me in … and said, “Do you reckon you could do this job?” I said, “I’ll give it a go,” and that was kind of it (CNC 2).*

At other times, they saw secondment opportunities as political and personal.

*There was a position and somebody would do it … and somebody else would do it and I remember thinking, I could do that but I can’t remember that being advertised (CNC 31).*

*You’ve got to be given that opportunity to start off with. You’ve got to be tapped on the shoulder* (*CNS 13).*

Other nurses felt there was a lack of support and saw this as an institutional system problem rather than a direct personal barrier.

*I asked my manager if they would support my application … and they said, no, they couldn’t do it because they would have to ask the next manager up (CNS 13).*

Alternatively, they saw the process of applying as just too hard. CNS (15) commented *some of them said, oh, it’s too much paperwork; the application is very – because it was - cumbersome.* From these descriptions, it is clear that the pathways to advanced practice positions are complex and it is difficult to be prescriptive.

### Changing jobs for career progression

With the complexity of health funding and the accreditation of limited positions, many of the participants, particularly those progressing into CNC or NP roles, discussed the merit of moving to other hospitals for advancement. Discussing how she could get a CNC role, CNS 13 commented: *I could probably travel … But yeah, I would have to go out of the area to get that.*

The constraints of travel or family responsibilities prohibited many nurses moving to progress their careers. CNS 20 commented:

*I would consider it, yeah … I’d have to commute, there’s nothing, no CNC roles at (hospital X)*

*… then this role as a CNS 2 came up about two and a half years ago and I was a little bit reluctant, because it’s a bit of a drive (CNS 4).*

### Self-driven

A common theme expressed by the CNS participants was that they needed to be self-driven for role progression as often there was little institutional or management support. CNS 3 commented:

… *have the time and the finances to do it, but then you've got to have the will to do it and then you've got to have the support of management and then your co-workers and things like that (CNS 3).*

*…the hoops that people have to go through to get study leave, it is really not encouraged to progress and to do it (CNS 14).*

Being self-driven to pursue education to increase their skills and knowledge was evident in that often these nurses used their annual leave to go to conferences or undertake studies. While others felt they were not going to pursue education if they had to finance it themselves. This is illustrated in CNS14 comment on other staffs’ lack of motivation to go to conferences if not supported.

*…they weren’t allowed to go to the conference. They could only go if they went in their own time. So they said, “Well, I’m not going to give up my holidays then”.*

There was also an understanding of the institutional challenges of financially supporting all staff with external studies and conferences.

*They do encourage us and they do want us to professionally develop, and I know that if they could, they would allow us to take as much study time as we needed (CNC 17).*

The main reason these nurses reported for attending conferences or undertaking further study was their self–motivation to continually improve their own knowledge and skill to ensure the delivery of evidence-based best practice and high quality patient services. Despite little or no institutional support, there was a clear understanding from all participants that engaging in ongoing professional education was paramount. CNS (16) affirmed: *we just do … because we’re focused about ourselves and about doing such a good job.*

### Effects of institutional environment

The institutional environment had a clear influence on career progression for this group of clinical nurse experts. One of the most widely discussed issues across all roles and positions was the participant’s perception of the institutional constraints on their career pathway opportunities.

*Obviously if there were more positions that would mean that management felt supported and valued the career structure of nurses. So I think it comes down to … I think that the limited positions that are, like there’s one CNC at the hospital, for the whole area (CNS 15).*

The fiscal environment of the institution was a significant factor in pursuing or procuring more advanced expert roles. A number of participants, when discussing the various position descriptions, commented that there was a lot of commonality and overlap in skills and functions of the CNS and CNC roles. Many were sceptical that this reflected a true career pathway, rather they questioned it was a cost saving mechanism.

*CNS 2, I find that a lot of their clinical domains in the State Award are very similar to the domains of a CNC 1. And my suspicion is that it’s been purposely devised like that as a cost-saving initiative, because a CNC 1 will probably get paid a few hundred dollars more a week than what a CNS 2 would get paid. (CNC 17)*

They felt there was no formal recognition of doing higher duties or support to regrade their positions. At times a position was downgraded, as reported by CNC 32, *now, when people leave, their CNC leaves, they’re replaced by a CNS 2.*

Environmental factors in establishing new NP positions within the politics and fiscal restraints were evident in the NP’s experiences. Two of the most significant barriers to this position were: the role being established in their specialty and the level of education required to fulfil this accredited position. Many CNS and CNC participants indicated that they were not motivated to undertake the tertiary studies necessary to become an NP because there were no positions for them to apply for in their specialty area. Further, they would have political difficulties finding a medical doctor to support their studies, which is mandatory for accreditation.

*…well, I wouldn’t want to go that full way and then have nothing at the end. So I want to be sure that I would have support behind me if I'm going to go down that road* (CNC 6).

Other participants were fearful that the funding for these positions was unstable.

*…my plan as a nurse practitioner… two years ago they did advertise the position. That was earlier on in my career and I wasn’t ready for the role but that has just, sort of, fallen over now (CNS20).*

While the environment could influence the establishment of NP roles, the NPs were adamant that the cycle of setting goals and appraising them during their career was essential. The NPs offered different advice regarding career progression. Rather than waiting for an established or advertised positions to be available, they suggested nurses find the specialty they were committed to and then pursue the necessary educational and clinical opportunities. They encouraged their peers to strive for the NP role and suggested nurses accept any opportunities offered to enable them to succeed.

*So don't waste your time doing courses or qualifications that are not going to help you reach an end point. You sort of have to be a little bit around the other way do the course and then start looking at your choices and opportunities (NP 25).*

## Discussion

This study has shown that many of the participants’ careers had been shaped haphazardly or serendipitously. In the main, the participants in this study did not elaborate on the two key motivators, professional empowerment and job satisfaction, which are postulated in the literature [12, 15, 18–20]. Many nurses moved into positions because of secondments determined by management. However, these secondments, which in the main were for CNS working in CNC roles, allowed the nurses to broaden their experience beyond bedside care and this had been recommended as a way to assist nurses in career development [12]. The nurses stated that being self-driven was a key to successful career achievement. As suggested by Cooper [27], setting milestones is key to successful career development, and as proposed by Donner and Wheeler [15] individual drive is essential in assessing and planning one’s career. In this study, the NPs appeared to be more career conscious than the CNSs and CNCs whose careers appeared somewhat unplanned.

Most of the participants were in their current roles for long periods, suggesting, as indicated by their age range, that their lack of drive to move up the career ladder could be a result of the fact that they were in the ‘harvest phase’ of their career [22]. In the NP cases, their positions were relatively new in the health system so they had more impetus to be more career directed with most staying in one specialty, suggesting they had aligned their personal strengths with their passions [21]. A second reason why the nurses, particularly those at CNS and CNC levels, were often not self-driven to develop a career pathway is because they either had to move or travel to gain promotion. This was because of the health system where new CNC and NP positions are determined by the state Ministry, which controls the financial situation in their organisation. This reflects the impact of the social and political environment of the workplace on individual career planning [28]. It also reflects that the participants are working in a closed system of career development where the forces that determined positions and appointments were the organisation where they worked [11].

Moving to an open system where nurses’ self-appraisal is included in career development is essential, which supports the iterative approach to career development advocated by Donner and Wheeler [15]. The program undertaken by Hall et al. [24] found that allowing nurses to self-appraise their career goals increased their work satisfaction and promoted a sense of empowerment. They recognised that nurses need support to understand the personal process of career planning, including undertaking a realistic self-assessment and thinking strategically. This could include acknowledging their demands outside work, an issue discussed by the participants in the current study. The environmental and political context also needs to be considered during the career planning process. Programs such as those developed by Duffield et al. [12] where clinical nurses were given time out to develop clinical and research portfolios were found to have a positive effect on career planning. This program, however, required financial support to backfill the nurses’ positions when away from the clinical area. Fiscal constraints in the health system means programs such as this are often difficult to implement.

Mentoring has been advocated as a way of assisting nurses in various aspects of their practice, but few studies have evaluated this approach for advanced practice nurses’ career development [14, 23]. One consideration when introducing mentoring to older nurses would be to ensure they felt their expertise and wisdom was appreciated. This requires special proficiency on the part of management and the mentors. Any mentoring relationship needs to be focused on assisting nurses to develop plans for their future careers rather seconding nurses into senior positions which is often at the convenience of the organisation. These secondments are frequently seen as mentoring by the organisation. Peer coaching could offer an additional or alternate approach to career development, where two or more nurses, at a similar grade or level, share their experiences [26]. While recognising that all interpersonal relationships can have challenges, peer coaching can improve participants’ self esteem, enable the acquisition of new skills, and engender a re-connection stimulated by the mutual dialogue. This may help the nurses to work with management in developing pathways for future clinical nurses.

## Implications for nursing

There are significant issues that need to be addressed by nursing management that go beyond the boundaries of the present study. Nursing management must encourage and support nurses to develop career plans and assist nurses by providing incentives, such as time release for education and programs that help them develop their professional potential. Additionally, in-house programs that assist nurses to address the challenges of career planning need to be developed and implemented, with the nurses providing input following a process of structured self appraisal of their abilities and developmental needs. It may be time for organisations to develop a clear descripton of mentorship and re-evaluate the meaning of this for nurses, who are striving to become experts in an environment that is often not supportive because of financial and organisational constraints. Exploration of other structured supportive approaches to career planning, such as peer coaching, may provide semi-formal strategies that could suit advanced practice nurses in the workforce. Further, unless knowledgeable and experienced nurses are given clear career pathways and opportunities, they may be lost to the profession. Therefore, the knowledge gained from this description of how a small group of nurses in urban Australia tread a career pathway can be important for future nurses who want to enter advanced practice.

## Limitations of the study

As in all qualitative research, the sample was small and taken from only one health district. Additionally, more than one interviewer collected data. During data analysis, it was found that while all constructs were covered by the interviewers, some appeared to stress different aspects, despite briefing and debriefing before and during the data collection process. However, the data were rich and gave a vivid picture of the status quo.

## Conclusions and recommendations

This snapshot of treading the clinical career pathway has provided evidence that it is often difficult for nurses who are not self driven because of personal and organisational constraints. In the case of the participants in this study, which may not be reflected in the literature, many nurses in their harvest years are happy to stay in their current position. Another determining factor in not developing their career path is the Health Ministerial constraints on new positions. Nurse managers’ perspectives on the career incentives for nurses in the current study have been analysed and will form part of a future paper. More work is required to assist nursing management, health systems and government to provide additional incentives for nurses to proactively develop and plan their careers. The current team is working on a project to further develop the role descriptors for the CNC; however, research focused on CNS role descriptors is required as the boundaries between CNS and CNC role becomes unclear. An intervention study also needs to be implemented and evaluated to assist nurses to be more career minded and work towards improving patient/client outcomes in an ever changing health care system through competence and motivation. Without self empowerment, support and ongoing education, the next generation of nurses may also find career development and planning serendipitous and haphazard.

## Abbreviations

CNS: Clinical nurse specialist
CNC: Clinical nurse consultant
NP: Nurse practitioner
